# Inaccuracy of Thyroid to Background Uptake Ratio in Evaluating Technetium-99m-pertechnetate Thyroid Uptake and Establishing an Improved Algorithm

**DOI:** 10.22038/AOJNMB.2019.12734

**Published:** 2019

**Authors:** Changyin Wang, Yanfen Zhao, Ying Shen

**Affiliations:** 1 Department of Nuclear Medicine, Zhongnan Hospital of Wuhan University, Hubei, China; 2 Department of Applied Mathematics, School of Mathematics and Statistics of Wuhan University, Hubei, China

**Keywords:** Quantitative analysis, Sodium pertechnetate Tc-99m, Thyroid gland, Thyroid uptake, Uptake ratio of thyroid-to-background

## Abstract

**Objective(s):**

The aim of this study was to explore the accuracy of thyroid to background uptake ratio (UR) in the evaluation of ^99m^Tc-pertechnetate thyroid uptake (TcTU) and establishment of an improved algorithm.

**Methods:**

This study was conducted on the thyroid images of 322 patients with thyroid diseases and 67 controls. For the purpose of the study, URs of the images were calculated, and then corrected by standardized thyroid area size to establish a corrected uptake ratio (CUR). Subsequently, the accuracy between UR and CUR was compared.

**Results:**

The results of linear regression using weighted least squares (using TcTU as a dependent variable and CUR, UR, or thyroid area size as independent variables) showed that CUR (t=105.5, P=0.000), UR (t=31.9, P=0.000), and thyroid area size (t=15.9, P=0.000) are influential factors of TcTU. Furthermore, the standardized coefficient of CUR (β=0.983) was obviously higher than those of UR (β=0.851) and thyroid area size (β=0.629). The linear goodness-of-fit between CUR and TcTU (R=0.983) was better than that between UR and TcTU (R=0.851). In addition, the total concordance rate between CUR and TcTU (96.7%) was significantly higher than that between UR and TcTU (83.0%; χ^2^=42.9, P=0.000). Discordance rates of CUR in large thyroid area (1.4% vs. 13.4%, χ^2^=17.0, P=0.000) and small thyroid area (3.3% vs. 42.2 %, χ^2^=44.3, P=0.000), were significantly lower than that of UR. In the abnormal thyroid areas, the discordance rates of UR obviously increased as compared to those of CUR. The UR overestimated the thyroid uptake in small thyroid areas and underestimated it in large thyroid areas.

**Conclusion:**

Based on the findings, CUR is more accurate than UR in measuring ^99m^TcO_4_ˉ thyroid uptake; accordingly, it is more significant in the diagnosis of thyroid disease.

## Introduction

^99m^Tc-pertechnetate thyroid uptake (TcTU) rate is a quantitative parameter used for various medical purposes, such as diagnosis (1-3), differential diagnosis (4), and evaluation of the therapeutic effects (5-7) of thyroid diseases. The TcTU is an accurate method in evaluating ^99m^TcO_4_ˉ thyroid uptake; however, this method is not widely used in clinical practices due to its relative complexity. 

Many semiquantitative methods, such as thyroid to thigh uptake ratio (8-10), thyroid to salivary ratio (11-14), thyroid to right upper lung ratio (15), thyroid to neck ratio (16), thyroid to mediastinum ratio, and thyroid to brain ratio, are more convenient than TcTU and are widely used to indirectly measure ^99m^TcO_4_ˉ thyroid uptake. However, these ratios are not always consistent with their TcTU values. Zhang et al. (16) reported contradictory results by observing high ratios of thyroid to background but normal TcTU values in some patients with subtotal or partial thyroidectomy. The present study involved a clinically similar situation to that of Zhang.

This study targeted toward proposing a new ratio algorithm. The new ratio method is more accurate than the above-mentioned ratio methods, and its accuracy is very similar to that of TcTU.

## Methods

***Study population***

The study protocols were approved by the Ethics Committees of Zhongnan Hospital of Wuhan University, Hubei, China (No. 2014065); furthermore, informed consent was obtained from all individuals. This study was conducted on 389 individuals undergoing thyroid imaging and TcTU measurement, including 67 controls (i.e., 16 males and 51 females; age range: 20-63 years, mean age: 38.5±10.6 years) and 322 patients with definite thyroid disease (i.e., 68 males and 254 females; age range: 18-71 years, mean age: 41.7±14.2 years). 

The thyroid patients consisted of 79 cases with Graves hyperthyroidism, 40 cases with simple goiter, 28 cases with subacute thyroiditis, 41cases with hypothyroidism, 23 euthyroid patients of postoperation of Graves hyperthyroidism, 44 postoperative patients of thyroid nodules, 48 cases with thyroid nodules and 19 cases with Hashimoto’s disease. There was no toxic nodular goiter case in the Graves’ hyperthyroidism and thyroid nodule groups. The patients in the hypothyroidism group had no determinable thyroid nodule. If the patients with hypothyroidism had a determinable nodule, they entered into the thyroid nodule group. The diagnosis of thyroid diseases accorded with the relevant guidelines (17, 18).

The individuals in the control group, who were initially suspected of having thyroid diseases, were finally confirmed to have no thyroid disease by clinical palpation, examination of color Doppler ultrasound, and laboratory examinations. According to our routine requirement, none of the patients had taken iodine-containing drugs, such as cydiodine buccal tablets 2 weeks before thyroid imaging and TcTU measurements (19).

***Grouping of individuals***

In this study, TcTU was accepted as a standard method for evaluating ^99m^TcO_4_ˉ thyroid uptake. The thyroid to background uptake ratio (UR) is a semiquantitative parameter. We analyzed the distribution of discordance rate between the UR and TcTU results in determining the reduction, normality, or elevation of ^99m^TcO_4_ˉ thyroid uptake. It was found that the discordance of UR was related to the thyroid area size and presented mainly under the condition of abnormal size of the thyroid area. For the purpose of the study, UR was corrected based on the average thyroid area size of the control group. Subsequently, the corrected uptake ratio (CUR) of thyroid to background and UR were compared in terms of accuracy.

To analyze the discordance distribution and discordance type of UR, the individuals enrolled in this study were assigned into three groups according to the thyroid area size. The three groups included small thyroid area (thyroid area<16.5 cm^2^; n=90), normal thyroid area (16.5 cm^2^≤thyroid area size≤27.6 cm^2^; n=157), large thyroid area (thyroid area size >27.6 cm^2^; n=142). 

To calculate the concordance rates between UR (or CUR) and TcTU and the discordance rates of UR and CUR in different thyroid sizes and uptakes and compare the accuracy between UR and CUR, the individuals were also divided into three groups according to TcTU, UR, and CUR. These groups included decreased thyroid uptake (TcTU, UR, or CUR values <lower limits of reference ranges), normal thyroid uptake (lower limits of reference ranges ≤ TcTU, UR, or CUR values ≤ upper limits of reference ranges), and increased thyroid uptake (TcTU, UR, or CUR values >upper limits of reference ranges). The reference ranges of the above-mentioned parameters are presented in the result section. 

***Imaging method***

Gamma camera with a low-energy and high-resolution collimator (e-CAM; Siemens product, Hoffman Estates, Illinois, USA), dose calibrator (FT-3106; Shanghai Electronic Instrument Factory, China), and ^99^Mo-^99m^Tc generator (Beijing Atomic Transtech Services, China) were used in this study. The calibration factor between the radioactivity intensity, measured with a dose calibrator, and the radioactivity counts, measured by a gamma camera, were calculated according to the manual provided by the gamma camera manufacturer. 

The radioactivity of syringe containing radiopharmaceutical were separately measured by means of the dose calibrator before and after the intravenous injection of Na^99m^TcO_4_ of 74-185 MBq. Twenty minutes after the intravenous injection of Na^99m^TcO_4_, thyroid imaging was performed while placing the patients in a supine position with the head hyperextended using a horizontal detector with a matrix of 256×256 and zoom of 3.2 taking 5 min. After the completion of thyroid imaging, the injection site was examined. If the injection leakage was visible in the monitor, a leakage imaging was performed for 1 min using a matrix of 256×256 and zoom of 1.0.

***Image processing and region of interest*** (***ROI) drawing***

**Figure 1:**
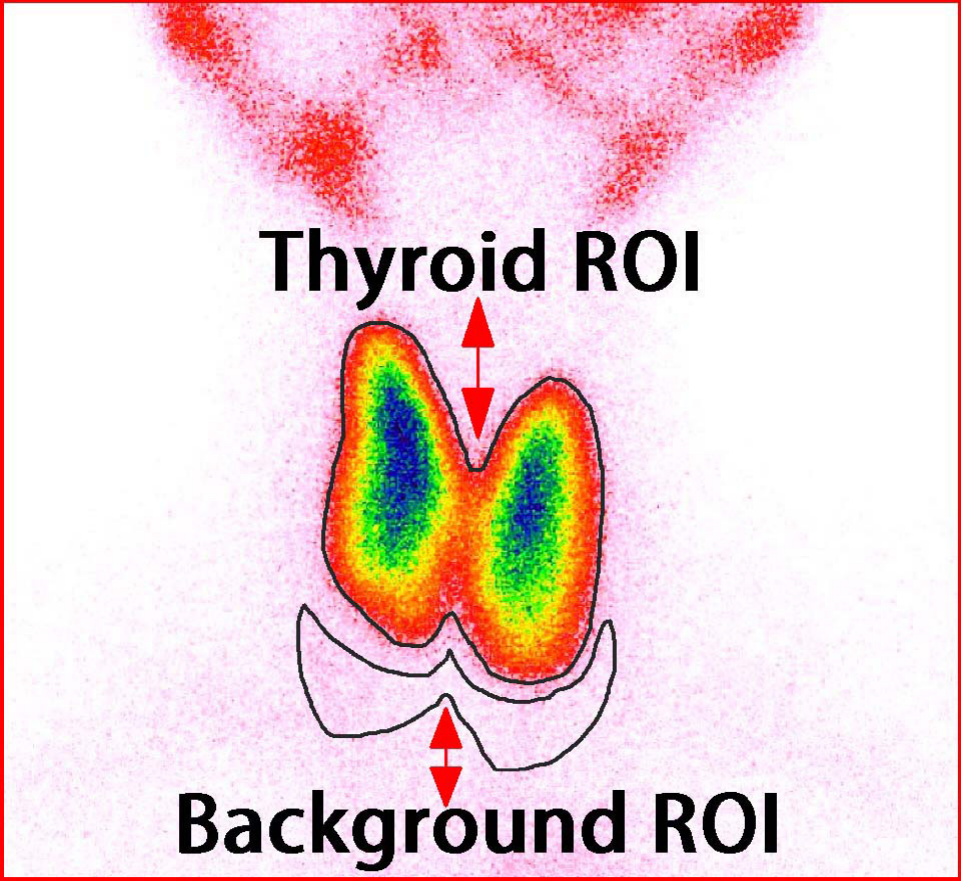
Schematic representation of the thyroid and background regions of interest

Thyroid images were displayed with a spectrum scale and a gamma parameter of 1.0. The contrast and brightness were adjusted to clearly show the edge of the thyroid tissue without altering the image size. The thyroid region of interest (ROI) was then outlined along the edge of the thyroid tissue while only surrounding the thyroid tissue with ^99m^TcO_4_ˉ uptake. The background ROI was outlined under the thyroid glands (Figure 1).

***Calculation of quantitative and semiquantitative parameters***

**Figure 2:**
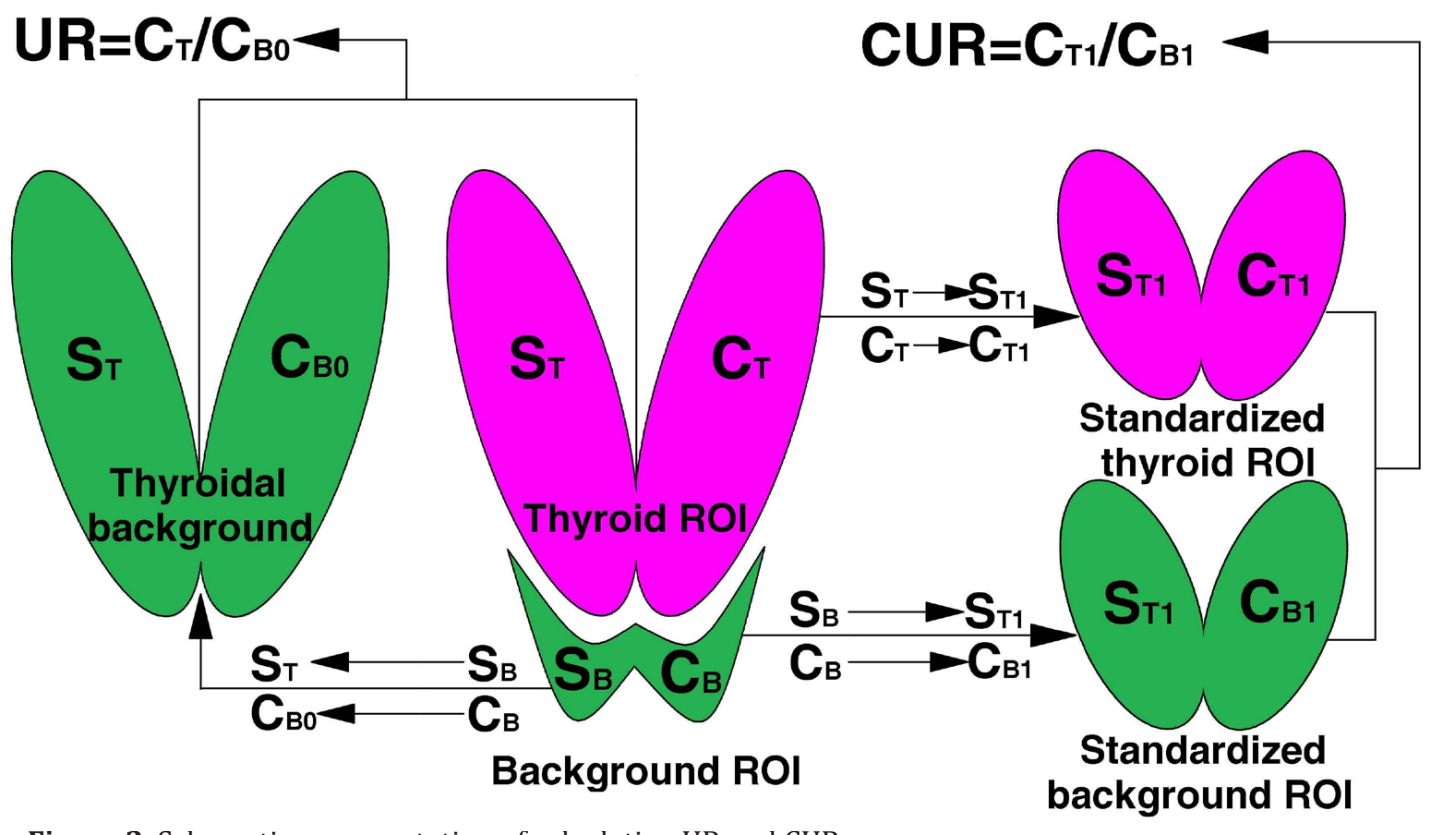
Schematic representation of calculating UR and CUR

Table 1 presents the formulae and parameters for calculating UR, CUR, and TcTU. The schematic representation of this procedure is also depicted in Figure 2. The specific algorithm of TcTU is given in the supplementary file 1. In these calculations, the average thyroid area of the control group was regarded as a reference standard. Then, the radioactivity counts in the thyroid ROI was converted into the radioactivity counts of the standardized size of thyroid (C_T1_). In addition, the radioactivity counts in the background ROI was converted into the counts in the standardized size of background (C_B1_).

***Statistical analysis***

The data were analyzed in SPSS statistical software (version 22.0; IBM Corporation, Armonk, New York, USA). The data are depicted in a scatterplot and listed in tables using median and interquartile range (IQR). Linear regression analysis by weighted least squares was used to evaluate the linear relationships between S_T_ and TcTU, between UR and TcTU, and between CUR and TcTU. In addition, the analysis of variance was adopted to determine the significance of the linear model. The significance of regression coefficients was also investigated by means of the t*-*test. Furthermore, the Chi-square test was performed to compare the difference in rates. Mann-Whitney U test as a nonparametric test was also used to compare the thyroid and control groups in terms of S_T_, TcTU, UR, and CUR. *P*-value less than 0.05 was considered statistically significant.

## Results

**Table 1:** Formulae and parameters for calculating ^99m^Tc-pertechnetate thyroid uptake rate, thyroid to background uptake ratio, and corrected thyroid to background uptake ratio

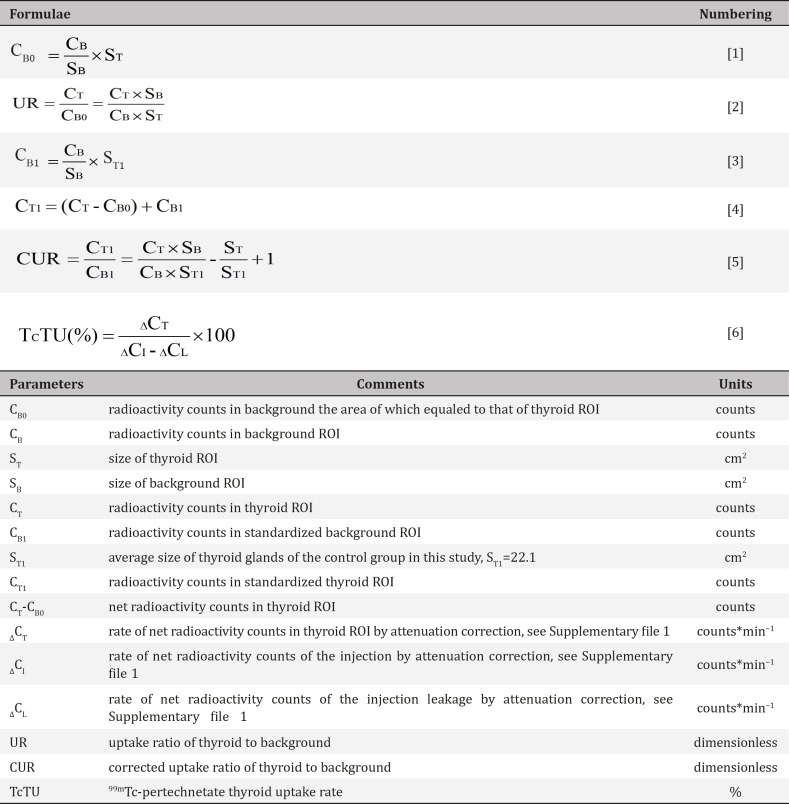

**Table 2:** Comparison of thyroid to background uptake ratio between thyroid disease groups and control group

**Group**	**n**	**M±IQR**	**Minimum**	**Maximum**	**Z**	**P**
Control	67	4.22±1.28	2.68	6.87		
Hyperthyroidism	79	14.37±9.32	5.49	33.79	-10.34	0.000
Simple goiter	40	4.16±2.55	2.52	18.52	-0.07	0.941
Subacute thyroiditis	28	1.59±0.43	1.19	2.71	-7.65	0.000
Hypothyroidism	41	1.99±2.60	1.07	24.47	-4.46	0.000
Postoperative hyperthyroidism	23	6.79±2.32	3.43	12.01	-5.62	0.000
Postoperative thyroid nodule	44	3.17±1.64	1.80	7.47	-3.62	0.000
Thyroid nodule	48	3.27±1.03	2.10	5.18	-4.97	0.000
Hashimoto’s disease	19	8.04±7.33	1.68	28.11	-1.59	0.111

M: median, IQR: interquartile range, UR: uptake ratio of thyroid to background

Mann-Whitney U test was used to compare the differences between groups with different thyroid diseases and control group in terms of UR.

**Table 3:** Comparison of corrected uptake ratio of thyroid to background between thyroid disease groups and control group

**Group**	**n**	**M±IQR**	**Minimum**	**Maximum**	**Z**	**P**
Control	67	4.03±1.45	2.49	6.35		
Hyperthyroidism	79	20.78±17.14	6.15	66.64	-10.39	0.000
Simple goiter	40	5.55±4.31	3.43	32.07	-4.93	0.000
Subacute thyroiditis	28	1.23±0.41	1.02	2.17	-7.66	0.000
Hypothyroidism	41	1.98±3.55	1.06	61.57	-3.73	0.000
Postoperative hyperthyroidism	23	3.62±2.29	1.98	6.43	-1.29	0.198
Postoperative thyroid nodule	44	2.03±1.28	1.09	6.62	-7.10	0.000
Thyroid nodule	48	3.87±1.13	2.13	5.51	-1.96	0.051
Hashimoto’s disease	19	7.22±7.73	1.68	45.56	-2.14	0.032

M: median, IQR: interquartile range, CUR: corrected uptake ratio of thyroid to background

Mann-Whitney U test was used was used to compare the differences between groups with different thyroid diseases and control group in terms of CUR.

**Table 4:** Comparison of accordance rates between UR to TcTU and CUR to TcTU in different thyroid area sizes

**Group**	**UR**		**CUR**		**χ** ^2^	**P value**
	**Case number**	**Rate (%)**	**Case number**	**Rate (%)**		
Decreased thyroid area size (*n*=90)	52	57.8^*^	87	96.7^**^	44.3	0.000
Normal thyroid area size (*n*=157)	148	94.3Δ	149	94.9^ΔΔ^	0.06	0.803
Increased thyroid area size (*n*=142)	123	86.6^▼^	140	98.6^▼▼^	17.0	0.000
All individuals (*n*=389)	323	83.0	376	96.7	42.9	0.000

Note: ▼ and *, *χ*^2^=24.4, *P*=0.000; Δ and▼, *χ*^2^=5.21, *P*=0.022; Δ and *, *χ*^2^=48.9, *P*=0.000; **, ΔΔ and▼▼, *χ*^2^=3.38, *P*=0.184.

TcTU:^ 99m^Tc-pertechnetate thyroid uptake rate, UR: thyroid to background uptake ratio, CUR: corrected thyroid to background uptake ratio

Chi-square test was performed to compare the rates.

**Table 5:** Goodness-of-fit of S_T_ to TcTU, UR to TcTU, and CUR to TcTU in a linear regression model using weighted least squares

**Dependent** **variable**	**Independent** **variable**	**R**	**Adjusted R** **square**	**Coefficients**				
				**b**	**Std. error of b**	**β**	**t**	**P-value**
TcTU	S_T_	0.629	0.394	0.143	0.009	0.629	15.93	0.000
	Constant			-0.330	0.037		-8.87	0.000
TcTU	UR	0.851	0.724	0.765	0.024	0.851	31.90	0.000
	Constant			-0.926	0.049		-18.74	0.000
TcTU	UR	0.894	0.799	0.708	0.021	0.788	33.74	0.000
	S_T_			0.024	0.002	0.282	12.09	0.000
	Constant			-1.267	0.051		-24.98	0.000
TcTU	UR*S_T_	0.982	0.964	0.028	0.000	0.982	102.61	0.000
	Constant			-0.294	0.020		-14.49	0.000
TcTU	CUR	0.983	0.966	0.680	0.006	0.983	105.52	0.000
	Constant			-0.738	0.017		-44.20	0.000
TcTU	CUR	0.975	0.952	0.667	0.016	0.974	42.55	0.000
	UR			0.001	0.021	0.001	0.06	0.955
	Constant			-0.711	0.021		-33.27	0.000
TcTU	CUR	0.983	0.966	0.685	0.007	0.990	94.12	0.000
	S_T_			-0.002	0.001	-0.015	-1.16	0.146
	Constant			-0.721	0.020		-35.44	0.000

TcTU: ^99m^Tc-pertechnetate thyroid uptake rate, UR: uptake ratio of thyroid to background, CUR: corrected UR, S_T_: thyroid area size, *R*: multiple correlation coefficient, *R* square: coefficient of determination,* b*: unstandardized regression coefficient, *β*: standardized regression coefficient

ROI: region of interest, S_B_: size of background ROI, S_T_: size of thyroid ROI, S_T1_: size of standardized thyroid ROI, C_B_: radioactivity counts in background ROI, C_B0_: radioactivity counts in background the area of which equaled to that of thyroid ROI, C_B1_: radioactivity counts in standardized background ROI, C_T_: radioactivity counts in thyroid ROI, C_T1_: radioactivity counts in standardized thyroid ROI, UR: thyroid to background uptake ratio, CUR: corrected thyroid to background uptake ratio

**Figure 3:**
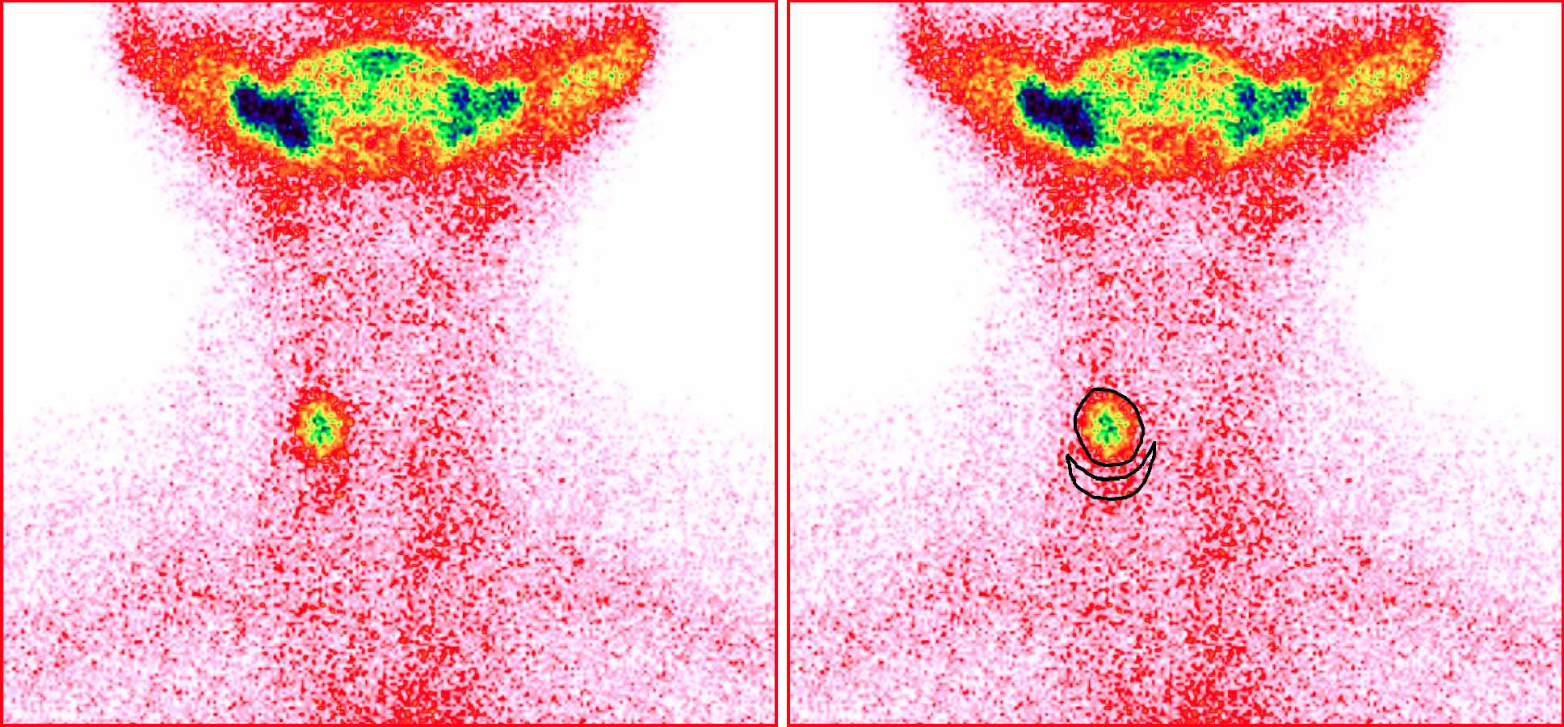
Thyroid imaging and ^99m^TcO_4_ˉ thyroid uptake in a 38-year-old female with subacute thyroiditis

(Only a partial tissue of the right lobe is visible. The TcTU was 0.07% [reference range: 0.82-3.52%] presenting obviously decreased ^99m^TcO_4_ˉ uptake, UR was 2.86 [reference range: 2.43-6.03] presenting normal ^99m^TcO_4_ˉ uptake, and CUR was 1.10 [reference range: 2.35-5.99], presenting obviously decreased ^99m^TcO_4_ˉ uptake. The results show that CUR is consistent with TcTU, but not with UR)

TcTU:^ 99m^Tc-pertechnetate thyroid uptake rate, UR: thyroid to background uptake ratio, CUR: corrected thyroid to background uptake ratio

**Figure 4:**
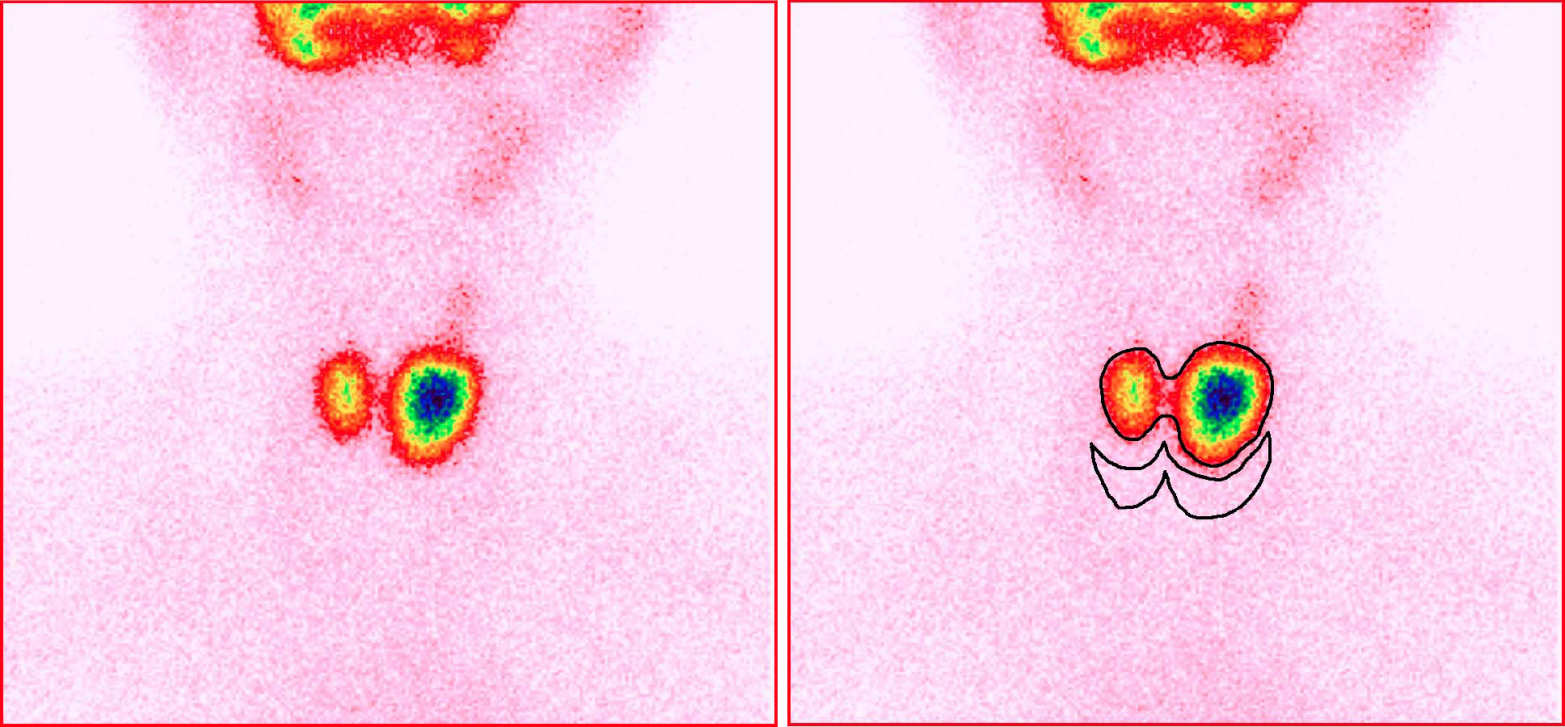
Thyroid imaging and ^99m^TcO_4_ˉ thyroid uptake in a 32-year-old female with euthyroidism after partial thyroidectomy

(Thyroid glands of this patient had been partially dissected because of Graves’ hyperthyroidism three years ago. Thyroid imaging reveals that the remaining thyroid [10.5 cm^2^] is smaller than the normal size [reference range: 16.5-27.6 cm^2^]. The TcTU was 2.37% [reference range: 0.82-3.52%] presenting normal ^99m^TcO_4_ˉ uptake, UR was 9.06 [reference range: 2.43-6.03] presenting increased ^99m^TcO_4_ˉ uptake, and CUR was 5.25 [reference range: 2.35-5.99] presenting normal ^99m^TcO_4_ˉ uptake. The results show that CUR is consistent with TcTU and the state of euthyroidism, but not with UR.)

TcTU:^ 99m^Tc-pertechnetate thyroid uptake rate, UR: thyroid to background uptake ratio, CUR: corrected thyroid to background uptake ratio

**Figure 5:**
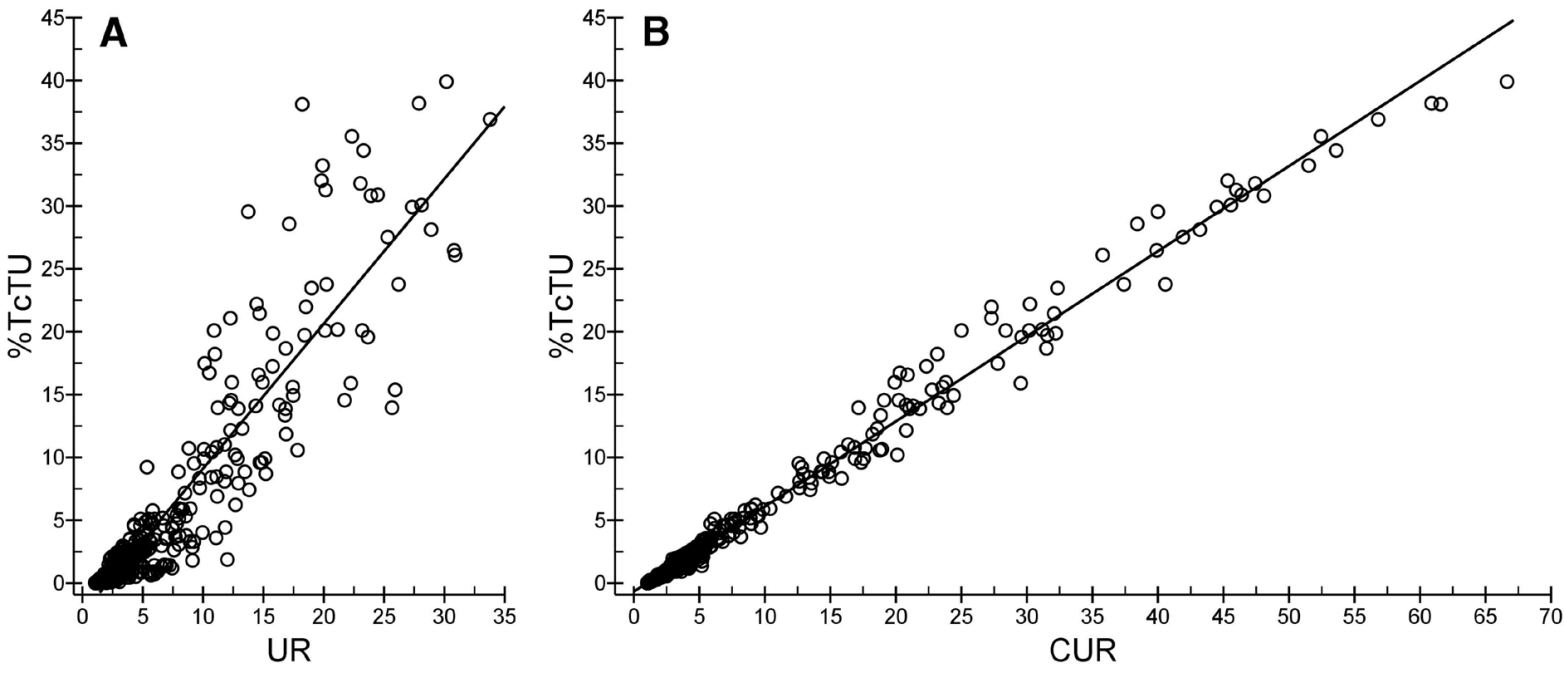
Comparison of the linear regression plots between UR to TcTU and CUR to TcTU;

A) scatterplot and linear fit line between UR and TcTU (*n*=389) and B) scatterplot and linear fit line between CUR and TcTU (*n*=389)

TcTU:^ 99m^Tc-pertechnetate thyroid up

***Reference ranges of TcTU, UR, CUR, and S***_T_

The mean TcTU, UR, CUR, and thyroid area size (cm^2^) were 2.17±0.69, 4.23±0.92, 4.17±0.93, and 22.06±2.82, respectively. The reference ranges were calculated at 95% confidence interval (mean±1.96SD). The reference ranges of TcTU (%), UR, CUR, and thyroid area size (cm^2^) were 0.82-3.52, 2.43-6.03, 2.35-5.99, and 16.5-27.6, respectively.

***Comparison of UR among thyroid and control groups***

The results of UR in patients with different thyroid diseases and control group are listed in Table 2. The results revealed that the UR of the Graves’ hyperthyroidism group was significantly higher than that of the control group (P<0.05). On the other hand, the URs of the patients with subacute thyroiditis, hypothyroidism, and postoperative thyroid nodule were significantly lower than that of the control group (P<0.05). 

In the four diseases, UR was similar to TcTU (Supplementary Table 1) in determining the thyroid uptake. The simple goiter and Hashimoto’s disease groups showed no significant difference with the control group in terms of UR (P>0.05), which is different from their TcTU. In this respect, the TcTU was higher in the simple goiter and Hashimoto’s disease groups than in the control group (Supplementary Table 1). 

The UR of the euthyroid patients after surgery for Graves’ hyperthyroidism was significantly higher than that of the control group (P<0.05). On the other hand, the TcTU of this group was significantly lower than that of the control group (Supplementary Table 1). Furthermore, the UR of the thyroid nodule group was significantly lower than that of the control group (P<0.05). However, there was no significant difference between these groups in TcTU (Supplementary Table 1). Therefore, UR obviously differed from TcTU in determining ^99m^TcO_4_ˉ thyroid uptake.

***Comparison of CUR among thyroid and control groups***

Table 3 summarizes the CUR results of patients with different thyroid diseases and control group. As the results indicated, the CURs of Graves’s hyperthyroidism, simple goiter, and Hashimoto’s disease groups were significantly higher than that of the control group (P<0.05). On the other hand, the CUR of subacute thyroiditis, Graves’ hypothyroidism, and postoperative thyroid nodule were significantly lower than that of the control group (P<0.05). The difference of CUR between the thyroid nodule and control groups was not statistically significant (P>0.05). 

In the seven diseases, CUR was similar to TcTU in determining pertechnetate thyroid uptake (Supplementary Table 1). The difference between the patients developing euthyroidism after surgery for hyperthyroidism and control groups was not significant in terms of CUR (P>0.05). However, TcTU was significantly lower in the euthyroid patients (occurring after hyperthyroidism operation) in comparison to that in the control group (Supplementary Table 1). Both CUR and TcTU were mostly in the normal range in this group and in line with their thyroid hormone level. In total, CUR was very similar to TcTU in determining ^99m^TcO_4_ˉ thyroid uptake.

***Concordance rates between UR and TcTU and between CUR and TcTU***

Table 4 presents the concordance rate between UR and TcTU and between CUR and TcTU in different thyroid sizes. The results showed that the concordance rate between UR and TcTU in small thyroid area was obviously lower than that in the large thyroid area (χ^2^=24.4, P=0.000). Furthermore, the concordance rate in large thyroid areas was significantly lower than that in normal thyroid area size (χ^2^=5.21, P=0.022). Therefore, in small thyroid areas, UR and TcTU had the lowest concordance rate. However, in different thyroid area sizes, the concordance rates between CUR and TcTU did not present a significant difference (χ^2^=3.38, P=0.184). In the abnormal thyroid area size, the concordance rates between CUR and TcTU were significantly higher than that between UR and TcTU (P<0.05; Table 4).

***Distribution of discordant UR results and comparison of discordance rate between UR and CUR***

Supplementary Table 2 lists the discordance rates of UR and CUR in different thyroid area sizes. According to the results, the discordant results of UR were mainly distributed in the small and large thyroid areas. The results showed that the UR discordance types were diverse. In the small thyroid areas, many patients with decreased TcTU were wrongly determined as normal ^99m^TcO_4_ˉ uptake by UR (Figure 3, Supplementary Figure 1), and many patients with normal TcTU were wrongly determined as high ^99m^TcO_4_ˉ uptake by UR (Figure 4). 

Furthermore, in the large thyroid areas, some patients with increased TcTU were wrongly determined as normal ^99m^TcO_4_ˉ uptake by UR, and some patients with normal TcTU were decided as low ^99m^TcO_4_ˉ uptake by UR. Under these conditions (i.e., small and large thyroid areas), the UR discordance rates were significantly higher than those of CUR (P<0.05). Nonetheless, in the normal thyroid area size, there was no significant difference between the CUR and UR in terms of the discordance rate (P>0.05).

***Comparison of the linear relationship between UR to TcTU and CUR to TcTU***

In order to explore the relationship between thyroid area size and TcTU and compare the linear relationship between UR to TcTU and CUR to TcTU, linear regression analyses by weighted least squares were performed between thyroid area size and TcTU, between UR and TcTU, and between CUR and TcTU. The results of regression analyses are presented in Table 5. The regression showed that thyroid area size and TcTU were linearly related (F=253.6, P=0.000), and 39.4% variation of TcTU could be interpreted by the thyroid area size. Linear regression also showed that UR and S_T_ presented a significant interaction. 

The UR*S_T_ is an interaction variable, and CUR is another type of the interaction variable. The results revealed CUR, UR, and thyroid area size as the influential factors of TcTU, respectively. However, when the UR and S_T_ were separately entered into the linear model, together with CUR, the variables of UR and S_T_ were not statistically significant (P>0.05). This indicates that CUR is a decisive factor as compared to the UR and S_T_.

As displayed in Figure 5, both CUR to TcTU and UR to TcTU presented a linear tendency. In Figure 5A, the points of UR to TcTU were distanced; however, in Figure 5B, the points of CUR to TcTU were very close. It was thus clear that the linearity of CUR to TcTU was better than that of UR to TcTU. The regression results confirmed that both UR to TcTU (F=1017.3, P=0.000) and CUR to TcTU (F=11134.5, P=0.000) were linearly related. The linear goodness-of-fit parameters of CUR to TcTU were higher than those of UR to TcTU (Table 5). According to the results, the correlativity between CUR and TcTU was obviously better than that between UR and TcTU. 

## Discussion

***Usefulness and limitations of UR***

The TcTU as an important quantitative parameter (20) and UR as a semiquantitative parameter, are the measurement methods of ^99m^TcO_4_ˉ thyroid uptake. In this study, the reference range of TcTU was obtained at 0.82-3.52%, which is similar to the ranges reported by Prakash (1.0-4.0%) in India (21), Zhang et al. (0.48-3.68%) in China (16), and Shigemasa et al. (0.7-3.0%) in Japan (22). However, it is markedly different from the reference range (0.35-1.7%) reported by Ramos et al. (23) and Zantut-Wittmann et al. (24) in Brazil. 

As indicated in the literature, the normal values of TcTU depend on the applied technique and dietary intake of iodide (8, 9). Therefore, each laboratory should establish its normal values. Zhang et al. (16) reported a strong correlation between semiquantitative ratio and TcTU (r=0.82, P<0.01). Given the simplicity of semiquantitative ratio, many different ratio methods are widely used for the evaluation of ^99m^TcO_4_ˉ thyroid uptake. The UR of this study is similar to the other semiquantitative ratios. 

Our results showed a linear relationship between UR and TcTU; furthermore, the total concordance rate between UR and TcTU was estimated at 83.0%, which is similar to the results reported in the literature (16). Therefore, UR can be used to estimate the ^99m^TcO_4_ˉ thyroid uptake of patients to some extents and is of important clinical value.

Despite its wide application, UR still has some shortcomings. Zhang et al. (16) found a discrepancy between UR and TcTU. Our study supported the viewpoint of Zhang et al. Under the condition of small thyroid area, 39.0% of the patients with decreased TcTU were wrongly determined to have a normal uptake of ^99m^TcO_4_ˉ (Supplementary Table 2, Figure 3, Supplementary Figure 1). In addition, 51.7% of the patients with normal TcTU were wrongly determined to have a high uptake of ^99m^TcO_4_ˉ (Figure 4, Supplementary Table 2), which showed that the quantity of ^99m^TcO_4_ˉ thyroid uptake was overestimated by UR. 

Under the condition of large thyroid area, 15.7% of the patients with increased TcTU were wrongly determined to have a normal uptake of ^99m^TcO_4_ˉ, and 12.0% of the patients with normal TcTU were wrongly determined to have a low uptake of ^99m^TcO_4_ˉ (Supplementary Table 2), which manifested that the quantity of ^99m^TcO_4_ˉ thyroid uptake was underestimated by UR. Therefore, UR presented much discordance under an abnormal thyroid size and could not completely replace TcTU.

***Why does UR present more discordance?***

The linear relationship between thyroid area size and TcTU indicated that the change of TcTU depends on the thyroid area size. Accordingly, the partial variations of TcTU may be explained by the change of the thyroid area size. Regarding this, thyroid area size is a factor influencing the accuracy of measuring ^99m^TcO_4_ˉ thyroid uptake by ratio methods. Changes in thyroid area size are common in patients with thyroid disease and after treatment for thyroid diseases (Supplementary Table 3). 

The UR is a relative ratio between the radioactivity in the thyroid and that in the background without a unit of measurement, which indicates that the relative uptake value does not include any information regarding the thyroid area size. Our findings demonstrated that the quantity of ^99m^TcO_4_ˉ thyroid uptake was affected by not only UR but also thyroid area size. Under an invariable UR condition, a bigger thyroid area size is accompanied by a higher quantity of ^99m^TcO_4_ˉ thyroid uptake. Based on this analysis, UR could not always accurately reflect the alteration of ^99m^TcO_4_ˉ thyroid uptake when disregarding the thyroid area size. 

After thyroidectomy, the area size of the residual thyroid tissues frequently becomes smaller and their UR could be similar to or higher than the preoperative UR given the influence of increased thyroid stimulating hormone. However, their absolute uptake of ^99m^TcO_4_ˉ after thyroidectomy is lower than that before thyroidectomy, resulting in occasional inconsistencies between UR and TcTU (Figure 4, Supplementary Figure 1). 

For subacute thyroiditis, under the condition of partial thyroid tissue imaging, the UR sometimes does not agree with TcTU (Figure 3). Meanwhile, our results showed that UR also had some discordance under a large thyroid area (13.4%). These phenomena demonstrate that the non consideration of thyroid area size is the main reason for the high discordance of UR under an abnormal thyroid area size.

***Methodological features of CUR***

Considering that the accuracy of UR in deciding thyroid uptake is dependent on the thyroid area size, we tried to correct UR in order to eliminate the influence of the thyroid area size. In the current study, the standardized size was determined based on the average thyroid area size of the control group and standardized thyroid area size of all individuals. 

Our results showed that the linear goodness-of-fit of CUR to TcTU was better than that of UR to TcTU, and the concordance rate between CUR and TcTU was significantly higher than that between UR and TcTU in the small and large thyroid areas. Therefore, the use of CUR as an evaluation parameter may obviously improve the accuracy of semiquantitative ratio in an abnormal thyroid area size. 

Furthermore, the results of a better linear relationship (R=0.983) and a higher concordance rate (96.7%) between CUR and TcTU showed that the accuracy of CUR was very similar to that of TcTU. Accordingly, CUR is a more accurate parameter in evaluating ^99m^TcO_4_ˉ thyroid uptake than UR. In a thyroid area of normal size, CUR and UR showed no significant difference in terms of concordance rates with TcTU. This indicates that UR is as highly accurate as CUR under this condition. Therefore, it is not necessary to correct UR under this condition. However, even though UR is corrected as CUR, its accuracy could not be decreased.

The CUR is a simplified semiquantitative parameter as UR. In the CUR formulae, S_T1_, denoting the mean thyroid area size of the control group, is a constant. The C_T_ and S_T_ of thyroid ROI, and C_B_ and S_B_ of the background ROI are unknown. As long as thyroid ROI and background ROI are outlined, the four unknown parameters are available. Then, CUR can be calculated according to the formulae. The calculation of CUR is simpler than the measurement of TcTU because it does not involve the measurement of the radioactivity of pre- and post-syringes by a dose calibrator, and the imaging of the pre- and post-syringes are performed by a gamma camera. 

Therefore, the use of CUR for evaluating thyroid uptake simplifies the operating process, decreases the workload of staff, and shortens the duration of working under gamma ray, thereby reducing the staff’s exposure to radiation in comparison to the measurement of TcTU.

***Clinical significance of CUR in thyroid diseases***

The CUR resembles TcTU and iodine-131 thyroid uptake rate; accordingly, it is an important value in evaluating thyroid diseases (1-7, 25, 26). Our results identified the consistent elevation of both CUR and thyroid hormones as the important feature of Graves’ hyperthyroidism. These features differ from those of subacute thyroiditis. Subacute thyroiditis is characterized by the elevation of the thyroid hormones and reduction of CUR (i.e., separation phenomenon). These features are similar to the results reported in the literature (2-4, 27). 

Graves’ hyperthyroidism could be efficiently discriminated from normal thyroid uptake by CUR or UR. However, the URs of some patients with subacute thyroiditis are normal, and sometimes, the UR fails to discriminate subacute thyroiditis from normal thyroid uptake. Our results indicated that CUR was more effective than UR in differentiating the low uptake of subacute thyroiditis from the normal thyroid uptake. Therefore, differentiating Graves’ hyperthyroidism and subacute thyroiditis is the most important application of CUR.

Thyroid uptake of Hashimoto’s disease and hypothyroidism presented complex features, their CUR showed different levels, namely reduction, normality, and even elevation. The levels of CUR in these diseases were not always consistent with their thyroid function levels. In this study, the CUR of simple goiter was either normal (55.0%; 22/40) or higher than the normal range (45.0%; 18/40), which shows that the decrease of CUR is not the feature of simple goiter. 

For Graves’ hyperthyroidism, both CUR and UR were consistently over the normal range. However, CUR and UR were normal in some patients with simple goiter. Therefore, the features of simple goiter were different from those of Graves’ hyperthyroidism. In the patients with simple goiter whose CUR was above the normal range, the UR of some patients was normal, which also differed from that of Graves’ hyperthyroidism. Under this condition, the combination of CUR with UR is valuable in differentiating simple goiter and Graves’ hyperthyroidism. When both CUR and UR increase, the differentiation between Graves’ hyperthyroidism and simple goiter must depend on their thyroid hormones.

Our results showed that it is sometimes impossible to accurately determine the ^99m^TcO_4_ˉ thyroid uptake of patients with partial thyroidectomy by means of UR. The patients with postoperative recurrent Graves’ hyperthyroidism had higher CUR and UR. In this study, 60.9% (14/23) of the patients with postoperative euthyroid also presented higher UR. As a result, it is difficult to differentiate these patients from those with postoperative recurrence by UR. 

However, the CURs of 92.9% (13/14) of the patients with increased UR were normal, and the CURs of only 4.3% (1/23) of the patients were slightly higher than the normal range. Therefore, CUR can accurately measure the ^99m^TcO_4_ˉ thyroid uptake of these postoperative patients. Accordingly, CUR is better than UR in differentiating between euthyroid patients and patients with postoperative recurrent Graves’ hyperthyroidism.

***Research Limitations***

The main limitation of this study was that the control group did not include minors; accordingly, it is not known whether the normal values of CUR and TcTU are applicable to minors (especially infants and young children). All of the selected individuals had at least one display of partial thyroid tissue. The patients without any visible thyroid tissue, such as diffuse subacute thyroiditis and total thyroidectomy, were not included in this study given the impossibility of outlining their thyroid ROI. However, the thyroid uptake level of ^99m^TcO_4_ˉ of this group was clear for the diagnostic purpose; therefore, the outlining of thyroid ROI was not necessary. 

Thyroid area size was defined as the size of functional thyroid tissue rather than the size of full thyroid area or thyroid volume. Therefore, thyroid area size can be only extracted from ^99m^Tc-pertechnetate thyroid imaging rather than from other nonfunctional imaging methods. The influence of thyroid thickness on ^99m^TcO_4_ˉ thyroid uptake was already reflected in UR; as a result, the UR correction using thyroid volume was not necessarily better than that using thyroid area size. Furthermore, this procedure is of relatively little value in diagnosing hypothyroidism. 

Radioiodine uptake test is generally preferred. Thyroid uptake is only an auxiliary diagnostic parameter. The diagnosis of thyroid disease is more effectively made by combining thyroid uptake and the measurements of serum thyroid hormones and thyroid stimulating hormone (TSH).

## Conclusion

The present study involved the comparison of the distribution of UR discordance results and analysis of the discordance type of UR results. As the findings indicated, the discordance results of UR were mainly distributed in small thyroid area, and the discordance types of UR results mainly included the overestimation of ^99m^TcO_4_ˉ thyroid uptake in small thyroid areas and its underestimation in large thyroid areas. 

After standardizing the thyroid area size of the patients, we compared the difference between CUR and UR. The results revealed that the concordance rate between CUR and TcTU and the linear relationship between CUR and TcTU were better than those between UR and TcTU. In other words, CUR is not only a simplified semiquantitative parameter but also a more accurate parameter in evaluating ^99m^TcO_4_ˉ thyroid uptake. Therefore, CUR is a valuable parameter in the clinical process of thyroid diseases.
